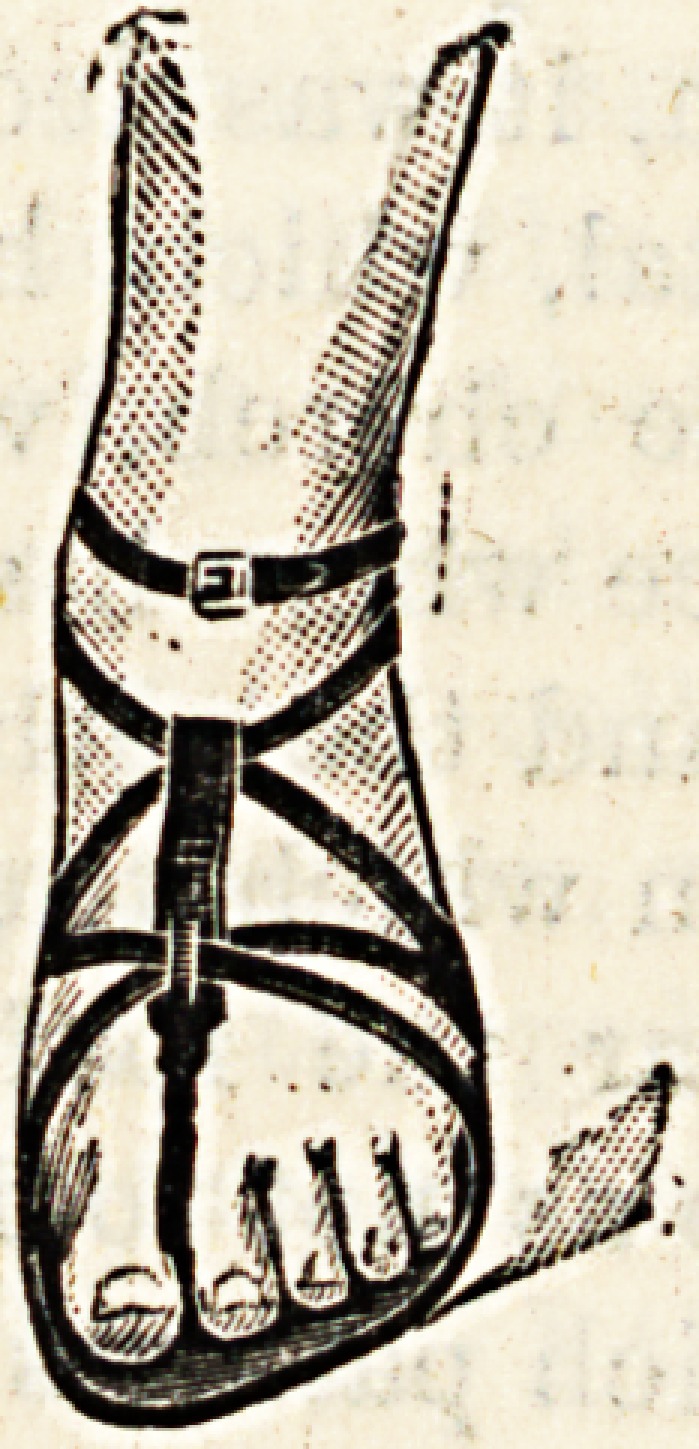# New Appliances and Things Medical

**Published:** 1901-07-20

**Authors:** 


### NEW APPLIANCES AND THINGS MEDICAL.

[We shall be glad to receive, at our Office, 28 & 29 Southampton Street Strand, London, W.C., from the manufacturers, specimens of all new preparations and appliances which may be brought out from time to time.]

### SANDALS FOR CHILDREN.

(LONDON SHOE COMPANY, LTD., 123 AND 125 QUEEN VICTORIA STREET, LONDON, E.C.)

THE accompanying illustrations represent a very practical form of sandal which can be obtained at the City Emporium of the London Shoe Company, or at their West End Depot, New Bond Street. The sandal appears a well manufactured article with a stout sole projecting well beyond the extremities of the toes, and the strap is well adapted for keeping the foot in position without chafing the skin. The leather though strong is soft and pliant. The advantages of sandals for children in hot weather are worthy of consideration. Not only are the feet and toes enabled to assume positions which are natural to them, but the free ventilation and exposure to sun and light which sandals permit are directly contributory to the general health.

### TAN SAN: A NEW MINERAL WATER.

(JAPANESE MINERAL WATERS SYNDICATE, 95 WIGMORE STREET, LONDON, W.)

WE have received a sample of this table water, which is bottled at the springs at Takaradzuka, near Kobé, in Japan. Although we have not submitted the water to a complete chemical analysis, it appears to be a water free from organic impurity, highly charged with carbonic acid gas, and possessing a sufficiently high mineral content to render the water crisp and refreshing to the taste.

## Figures and Tables

**Figure f1:**
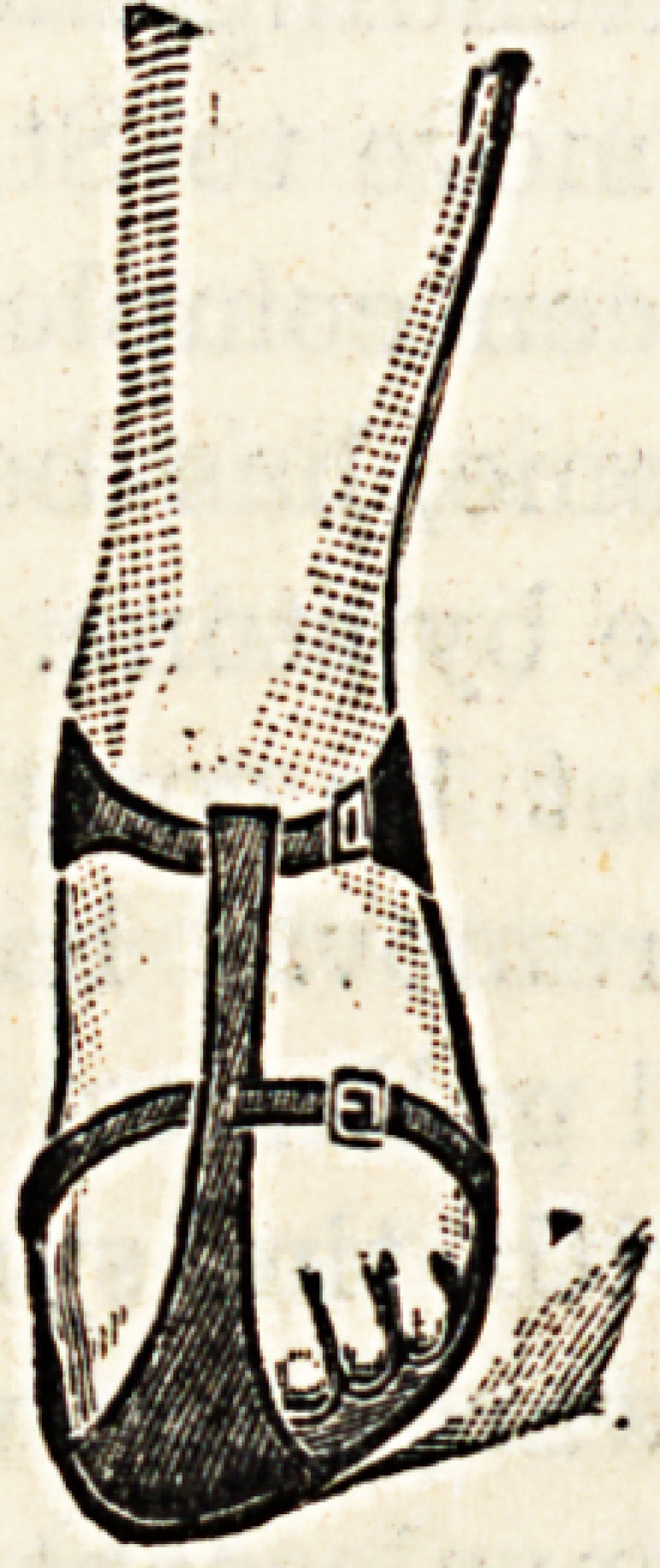


**Figure f2:**